# Biogeography of *Nocardiopsis* strains from hypersaline environments of Yunnan and Xinjiang Provinces, western China

**DOI:** 10.1038/srep13323

**Published:** 2015-08-20

**Authors:** Song-Tao He, Xiao-Yang Zhi, Hongchen Jiang, Ling-Ling Yang, Jin-Yuan Wu, Yong-Guang Zhang, Wael N. Hozzein, Wen-Jun Li

**Affiliations:** 1 State Key Laboratory of Biocontrol and Guangdong Key Laboratory of Plant Resources, College of Ecology and Evolution, Sun Yat-Sen University, Guangzhou, 510275, China; 2 Key Laboratory of Microbial Diversity in Southwest China, Ministry of Education, Yunnan Institute of Microbiology, Yunnan University, Kunming, 650091, China; 3 State Key Laboratory of Biogeology and Environmental Geology, China University of Geosciences, Wuhan 430074, China; 4 Key Laboratory of Biogeography and Bioresource in Arid Land, Chinese Academy of Science, Xinjiang Institute of Ecology and Geography, Chinese Academy of Sciences, Ürűmqi 830011, China; 5 Bioproducts Research Chair (BRC), College of Science, King Saud University, Riyadh 11451, Kingdom of Saudi Arabia

## Abstract

The genus *Nocardiopsis* is a widespread group within the phylum *Actinobacteria* and has been isolated from various salty environments worldwide. However, little is known about whether biogeography affects *Nocardiopsis* distribution in various hypersaline environments. Such information is essential for understanding the ecology of *Nocardiopsis*. Here we analyzed 16S rRNA, *gyrB*, *rpoB* and *sodA* genes of 78 *Nocardiopsis* strains isolated from hypersaline environments in Yunnan and Xinjiang Provinces of western China. The obtained *Nocardiopsis* strains were classified into five operational taxonomic units, each comprising location-specific phylo- and genotypes. Statistical analyses showed that spatial distance and environmental factors substantially influenced *Nocardiopsis* distribution in hypersaline environments: the former had stronger influence at large spatial scales, whereas the latter was more influential at small spatial scales.

The study of biogeography addresses spatial patterns of species and ecosystems in geographic space or through geological time^1^. It is widely accepted that macro-organism populations have many similar characteristics and common ancestry and thus possess different geographical distributions due to geographical isolation^2^. In contrast, debate has been lasting on microbial biogeography in that microorganisms have great potential for global dispersal and adaptability to diverse environments^3^. However, numerous studies recently reported bio-geographic patterns for microbial distributions^4^,^5^.

Microbial biogeography is mainly ascribed to two factors: the environmental heterogeneity and spatial distance^6^, either of which alone cannot entirely account for geographic distribution patterns of some widespread bacterial groups^7^,^8^. However, little is known about the relative importance of these two factors to shaping microbial biogeography in saline and hypersaline environments. Theoretically, saline and hypersaline environments are suitable for studying impact of environmental effects on bacterial biogeography because of strong environmental selective pressures in such environments^9^. Previous studies have shown that spatial distance and environmental factors contribute differently to shaping microbial biogeography in saline environments with respect to certain microbial groups^10^.

As one group of widespread Actinobacteria, *Nocardiopsis* species have drawn extensive attention from microbial ecologists due to their capacity to produce compounds of potential biotechnological relevance^11^,^12^,^13^. Most characterized *Nocardiopsis* strains were mainly recovered from various salty habitats, such as marine environments, deserts, alkaline or hypersaline soils^14^,^15^,^16^. In these salty environments, *Nocardiopsis* spp. underwent a wide range of environmental pressures and thus developed distinct genetic and metabolic features among different habitats^11^,^12^,^13^. So in order to understand the underlying reasons for the endemicity of *Nocardiopsis* species in various hypersaline environments, it is imperative to know 1) whether geographic isolation affects *Nocardiopsis* distribution in hypersaline environments and 2) relative importance of spatial distance and environmental factors to shaping *Nocardiopsis* distribution in (hyper) saline environments.

Investigation on the biogeography of *Nocardiopsis* spp. requires a detailed taxonomic classification of *Nocardiopsis* strains from different habitats. High phylogenetic resolution can be achieved by multilocus sequence analyses, which combine phylogenies of 16S rRNA and functional housekeeping genes such as *gyrB*, *rpoB* and *sodA*^17^,^18^,^19^. Previous studies have shown that 16S rRNA gene together with *gyrB*, *rpoB* and *sodA* genes could provide better phylogenetic resolution^20^,^21^,^22^,^23^ than one single gene. Here, we applied a multilocus phylogenetic analysis of 16S rRNA, *gyrB*, *rpoB* and *sodA* genes to investigate the biogeographic patterns of *Nocardiopsis* strains retrieved from the hypersaline environments in Xinjiang and Yunnan Provinces of western China and assessed their correlations with spatial distance and environmental factors.

## Results

### Geochemistry differentiations of the sampled sediments

In Xinjiang Province, Qijiaojing Salt Lake is 140 km away from Aydingkol Salt Lake; while in Yunnan Province, Heijing Salt Mine is about 560 km away from Jiangcheng Salt Mine. The sampling sites of Xinjiang Province are about 4300 km away from those of Yunnan Province (Table S1). The Heijing and Jiangcheng salt mines are under subtropical monsoon climate. The salt ores in the two salt mines are rich in sodium chloride as well as potassium chloride^24^. In Xinjiang Province, due to strong evaporation, most of surface areas of Aydingkol and Qijiaojing salt lakes have been highly mineralized, containing abundant alkaline rock salt (e.g., Glauber’s salt, glauberite, gypsum, sodium chloride) but low concentration of potassium salt^25^.

**Figure 1:**
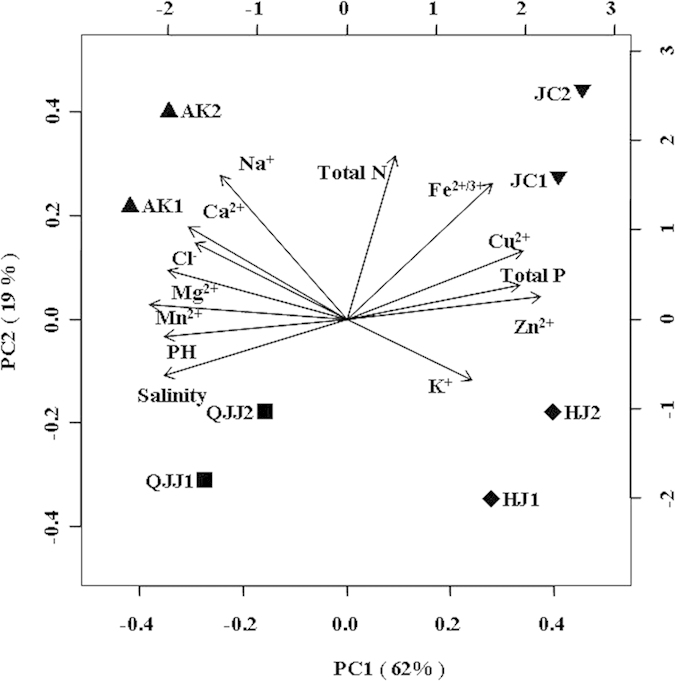
(**A**) PCA map showing the 8 sampling sites and their correlation with trace elements. Note: pH, Cl^–^, Ca^2+^, Mg^2+^, K^+^, Na^+^, Fe^2+^, Mn^2+^, Cu^2+^, Zn^2+^, total N (nitrogen) and total P (phosphorus) were used to evaluate the influence of each variable. The longer the arrow, the greater the influence; the smaller the angle between two arrows, the closer their correlation. solid squares (■), upright (▲) and inverse (▼) triangles, and diamonds (♦) denote the Qijiaojing (QJJ), Aydingkol (AK), Jiangcheng (JC) and Heijing (HJ) sampling sites, respectively.

Principle component analysis (PCA) showed that the Yunnan sampling sites were different from that of Xinjiang with respect to environmental factors: the sediment chemistry of the Aydingkol and Qijiaojing salt lakes was different from that of the Heijing and Jiangcheng salt mines: the former possesses higher salinity, pH and concentrations of Ca^2+^, Mg^2+^ and Mn^2+^ but lower concentrations of trace elements (*e.g.* K^+^, Cu^2+^, Zn^2+^) and total phosphorus than the latter (cumulative contribution value = 91.5%, Fig. 1). In addition, the sampling sites within one region (Yunnan or Xinjiang) were different from each other on the basis of climate types, geographic distances, and geochemistry factors (Tables S1 & S2, Fig. 1).

### Phylogenic analysis of the 16S rRNA, *gyrB*, *rpoB* and *sodA* genes

**Figure 2:**
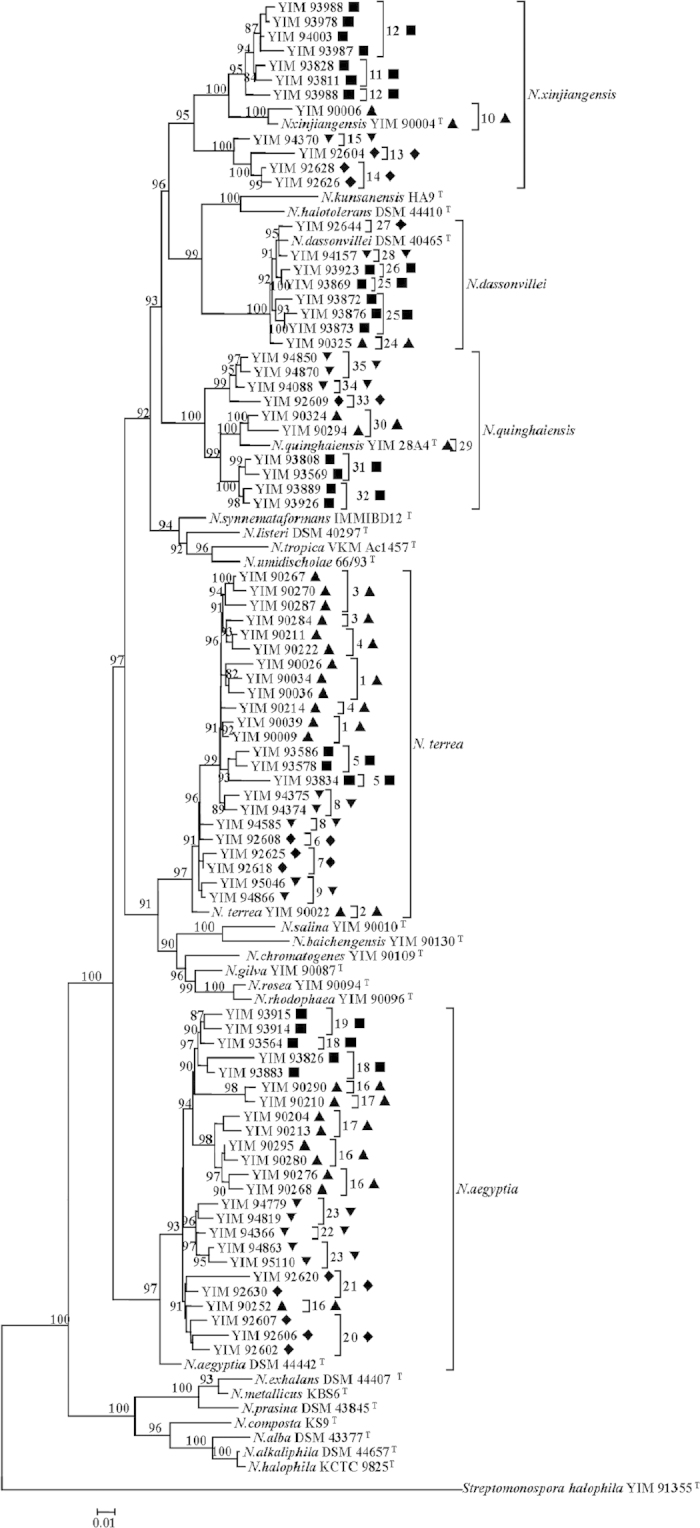
Maximum Likelihood based Phylogenetic tree of concatenated sequences of 16S rRNA, *gyrB*, *rpoB* and *sodA*, showing endemism for five *Nocardiopsis* OTUs. Bar, 0.05, five nucleotide substitutions per 100 nt. Bootstrap values are shown as percentage of 1000 replicates, and only the bootstrap values above 50% are shown. Solid squares (■), upright triangles (▲), inverse triangles (▼) and diamonds (♦) indicate *Nocardiopsis* strains from Qijiaojing (QJJ) and Aydingkol (AK) sampling sites of Xinjiang Province and Jiangcheng (JC) and Heijing (HJ) sampling sites of Yunnan Province, respectively. The 34 STs are marked in the clades, and each ST is supported by high bootstrap value (>80%).

A total of 78 *Nocardiopsis* strains were retrieved and subjected to phenotypic characterization as well as OTU identification^26^. The obtained *Nocardiopsis* strains belonged to five OTUs (*N. dassonvillei*, *N. aegyptia*, *N. terrea*, *N. quinghaiensis*, and *N. xinjiangensis*) (Table S3). Each of the identified OTUs covered more than eight strains and contained at least one strain from a sampling site (Table S3). The multi-locus sequence typing (MLST) phylogeny showed endemism of *Nocardiopsis* strains: each endemic sequence type (ST) was specific to a site or a region (Table 1 and Fig. 2 & Fig. S1E, Bootstrap value >80%). A total of 34 STs (Table S4) were identified, with either region containing 17 STs and each sampling site including at least 8 STs (Table 1 & Table S5).

### Effects of spatial distance and environmental factors on the geographic patterns of *Nocardiopsis* strains

**Figure 3:**
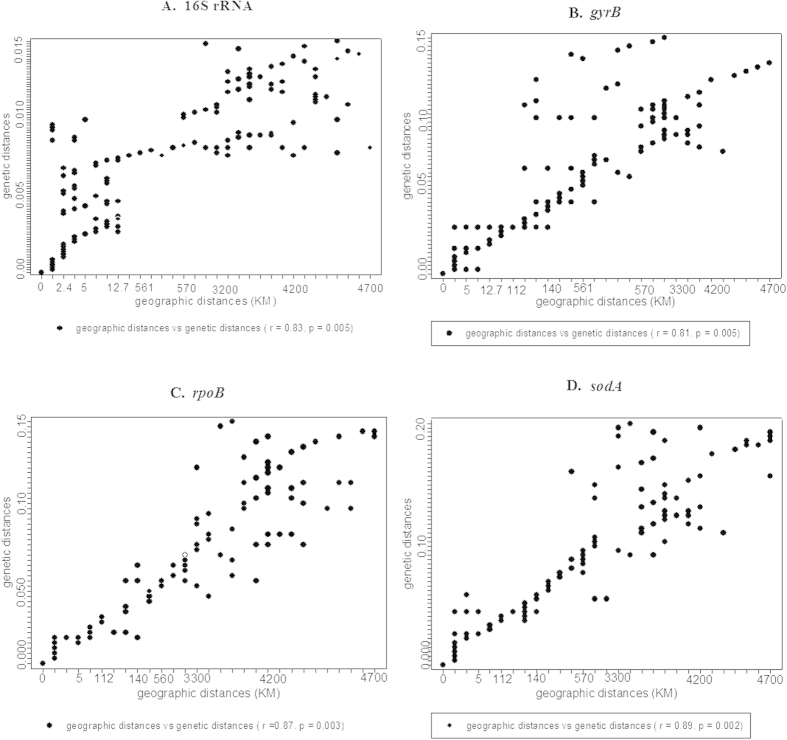
Mantel correlation between genetic distances of the four genes and geographic distance for the five identified *Nocardiopsis* OTUs. (**A–D**) panels indicate the correlation between geographic distance and genetic distances of 16S rRNA gene (r = 0.83, p = 0.005), *gyrB* (r = 0.81, p = 0.005), *rpoB* (r = 0.87, p = 0.003) and *sodA* (r = 0.89, p = 0.002) genes, respectively.

**Figure 4:**
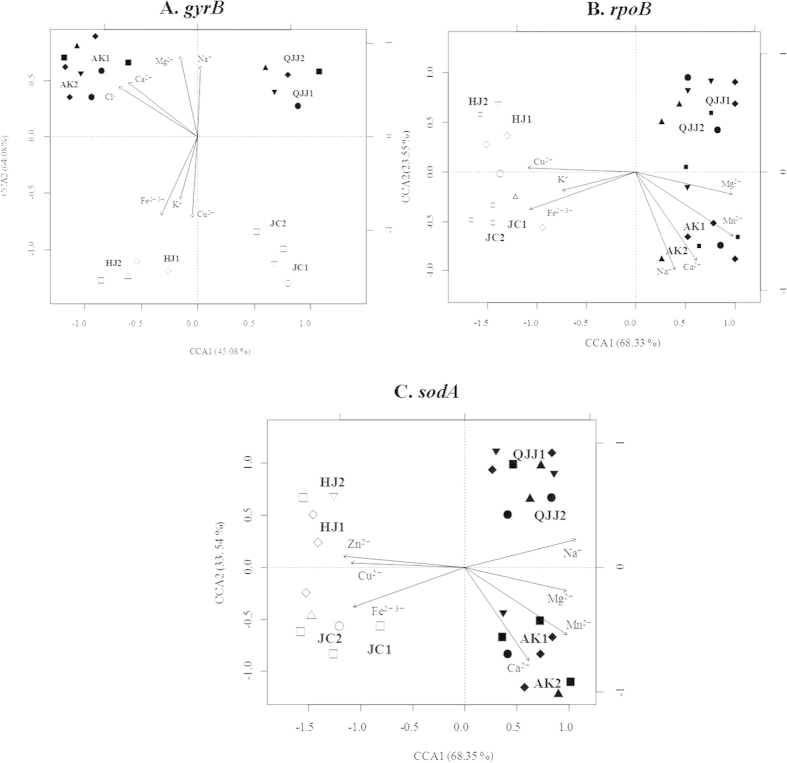
Canonical correlation analysis of linear relationships between geochemistry variations, and differentiations of endemic genotypes among the eight sampling sites (**A–C**) panels are for *gyrB*, *rpoB*, and *sodA* genes, respectively). In *Nocardiopsis xinjiangensis*, inverse solid (▼) and open (▽) triangles and upright solid triangles (▲) denote NXX, NXY and NDX phylotypes/genotypes from Xinjiang sites, respectively; upright open triangles denote NDY phylotypes/genotypes from Yunnan sites; in *Nocardiopsis quinghaiensis*, solid (●) and open (◯) circles denote NQX and NQY phylotypes/genotypes from Xinjiang and Yunnan sites, respectively; in *Nocardiopsis aegyptia*, Solid (■) and open (□) squares denote NAX and NAY phylotypes/genotypes from Xinjiang and Yunnan sites, respectively; in *Nocardiopsis terrae*, solid (◆) and open (◇) diamonds denote NTX and NTY phylotypes/genotypes from Xinjiang and Yunnan sites, respectively.

MLST of four housekeeping genes revealed significant correlations of gene sequences with geographic distance (16S rRNA: r = 0.83, p = 0.005; *gyrB*: r = 0.81, p = 0.005; *rpoB*: r = 0.87, p = 0.003; *sodA*: r = 0.89, p =0.002) (Fig. 3). In addition, the closely related phylo- or genotypes were present within a very small scale (<100 km) but not at distant locations (>100 km) (Fig. 3). Mantel test (r > 0.5, *P* < 0.05) and canonical correlation analysis showed that the differentiations of endemic genotypes of *gyrB*, *rpoB* and *sodA* genes were significantly correlated with the geochemistry variations of sediments from eight habitats between Yunnan and Xinjiang Provinces (Table 2; Fig. 4).

The Z-test of three functional housekeeping gene (*gyrB*, *rpoB* and *sodA*) transcripts by optimum ‘positive selection’ models (M2a and M8) showed that evolutions of *gyrB*, *rpoB* and *sodA* genes in the 78 *Nocardiopsis* strains in this study and 24 type strains from other habitats were partially under positive environmental selection (ω_2_  1 and ω_8_  1, respectively, Table S6). Mutations of seven, five and five residues of the B subunit of DNA gyrase, the *β* subunit of RNA polymerase and the A subunit of superoxide dismutase, respectively, were positively influenced by environmental forces (Table S6). The predicted molecular function ontology of *gyrB*, *rpoB* and *sodA* gene transcripts (Table S7) showed that seven N-terminus residues of the B subunit of DNA gyrase had high potential for binding magnesium ion, ATP or integrating with nucleotides, five N-terminus residues of the *β* subunit of RNA polymerase had high potential of binding cations or rifampicin, and five N-terminus residues of the A subunit of superoxide dismutase had high potential of binding magnesium, iron, or copper ions.

## Discussion

### Geographic patterns and endemism of *Nocardiopsis* genotypes within an OTU

Our study supports previously detected biogeographical patterns among *Nocardiopsis*, being consistent with the fact that some *Nocardiopsis* species have been exclusively isolated from certain habitats to date^27^,^28^,^29^,^30^,^31^. The patchy distribution of *Nocardiopsis* among species and endemic patterns within one species (Fig. 2 & Fig. S1E) indicated that biogeography may influence microbial distribution within a species but may not function among species within the genus at a large geographic scale. The observed endemic distribution of *Nocardiopsis* strains was consistent with previous studies about other microbial groups^32^,^33^,^34^,^35^,^36^,^37^. For example, a patchy geographic distribution was found for the bacterial isolates within a homogeneous background (sulfate-reducing sediments from four continents^32^. Similarly, crenarchaeal assemblages in mesophilic soil habitats were distributed in mosaic patterns of different phylotypes^6^,^36^. Likewise, individual genotypes of purple non-sulfur bacterium *Rhodopseudomonas palustris* were detected only locally and exhibited patchy distribution at 10-m or even 1-m scales^33^.

### Relative importance of spatial distance and environmental factors upon endemism of *Nocardiopsis* strains

The biogeographic distribution of *Nocardiopsis* could be ascribed to spatial distance and environmental factors. However, little is known about the relative importance of spatial distance and environmental factors on the distributional patterns of the five known *Nocardiopsis* species. In this study, the impact of spatial distance upon endemicity of *Nocardiopsis* in Yunnan or Xinjiang could be validated by the fact that the closely related phylo- or genotypes was present within a very small scale (<100 km) but not at distant (>100 km) locations (r > 0.80 p < =0.005, Fig. 3). This indicated that spatial distance significantly contribute to the observed biogeographic patterns of *Nocardiopsis* strains at a large scale, which was consistent with some previous studies^6^,^38^. Previously, spatial distance together with genetic drift or physical isolation was proposed to lead to microbial population endemism at a large scale^38^,^39^. Our data suggest that the spatial distance notably resulted in differentiations of *Nocardiopsis* strains between regions (>100 km, Fig. 3).

Our study suggested that both environmental parameters and spatial distance played a role. However, environmental parameters apparently rather influence microbial endemism at the local scale. In the present study, the genetic differentiations of *gyrB*, *rpoB* and *sodA* genes of the retrieved *Nocardiopsis* strains significantly corresponded to heterogeneities of some cations or anions in the sediments of the studied sampling sites within a habitat (Table 2 and Fig. 4). This observation was consistent with some previous studies, in which environmental factors rather than spatial distance were shown to cause bacterial variation at a local scale (within 1 km)^38^,^39^. Previous studies indicated that Na^+^, Mg^2+^, Ca^2+^, Mn^2+^ and Fe^2+/3+^ ions were significant in influencing bacterial biogeography at the species (97% OTU) or subspecies (99%) levels^40^. The Na^+^ ions were important to some halophilic bacteria or alkaliphilic bacteria as they replaced protons and coupled ion to cope with the high external pH, rather than increasing the electric potential difference across the cytoplasmic membrane^41^. Mg^2+^ was a chaotropic agent and a limiting factor in the diversity of microbes in the hypersaline environment^39^. In addition, Cu^2+^, Ca^2+^, Mn^2+^ and Fe^2+/3+^ ions were important regulators of some extremozymes in *Nocardiopsis* genus, for example, xylanases, alpha amylases, thermoalklotolerant *β*-1,3-glucanases and cellulases^42^,^43^. Thus, it is reasonable to observe the significant influence of environmental factors on the biogeographic distribution of *Nocardiopsis* strains.

Environmental factors influenced some functional genes important for bacterial survival more significantly than 16S rRNA gene. For example, the phylogenies of *gyrB*, *rpoB* and *sodA* genes of the obtained *Nocardiopsis* strains showed more visible endemic clusters within one habitat or one region than the highly conserved 16S rRNA gene (Fig. 2 & Fig. S1, Table 1). This observation could be ascribed to the fact that some residues of the three functional housekeeping genes could be subjected to mutation due to cation binding (*P* > 90%, Table S6 and Table S7; reliability >70%), which led to the catalytic regulation functions of their corresponding enzymes^44^,^45^,^46^,^47^,^48^.

In summary, *Nocardiopsis* spp. in hypersaline environments possessed geographic distribution patterns. Spatial distance and environmental factors influenced the biogeography distribution of *Nocardiopsis* at large and local scales, respectively.

## Material and Methods

### Site description and sample collection

In this study, two salt mines (Heijing and Jiangcheng) from Yunnan Province and two salt lakes (Aydingkol and Qijiaojing) from Xinjiang Province of western China were selected (Table 1). Two sites each were sampled at the Heijing saline mine (HJ1, an abandoned salt mine; HJ2, a natural hypersaline spring) and the Jiangcheng salt mine (JC1, an abandoned salt mine; JC2 site, a natural hypersaline spring), respectively. Two (AK1 and AK2) and two sites (QJJ1 and QJJ2) were sampled at Aydingkol and Qijiaojing salt lakes, respectively. At each selected sampling site, sediments were sampled at the 10–30 cm depth and collected into sterile 50 ml sterile Falcon centrifuge tubes. GPS coordinates were recorded at each sampling point with a portable meter in the field and were subsequently imported into Map-Source according to the manufacturer’s instructions to measure the geographic distances among the sites. The samples for microbial cultivation and geochemistry measurement were stored at 4 °C in the field and during transportation.

### Geochemistry measurements

The pH and salinity of the sampled sediments were measured with portable meters after sediments being dissolved into distilled water. The concentrations of major cations and trace elements in sediments from nine sampling sites were measured by flame atomic absorption spectrometry (HITACHI Z-2310). Total nitrogen of the sediment samples was determined by the semi-micro-Kjeldahl method^49^, and total phosphorus of the sediment samples was determined by the alkali fusion–Mo-Sb Anti-spectrophotometric method^49^. Principle component analysis (PCA) of the studied sediment samples was performed with the use of the R program^50^.

### Isolation of *Nocardiopsis* strains

The sediment samples (2 g, wet weight) were dispersed into 18 ml sterilized physiological saline water (con. 0.70%, w/v, equal to bacterial cell physiological salinity) and were incubated at 30 °C for 30 min with shaking at 150 rpm. The resulting slurry was serially diluted with sterilized physiological saline water (NaCl con. 0.70%, w/v). Aliquots (0.2 ml) of each dilution were spread onto petri dishes containing three different media: cellulose-casein multi-salt medium and modified ISP 4 and ISP 5 media^51^. All the agar plates were supplemented with 5% (w/v) NaCl and potassium dichromate (15 mg/L)^51^. The petri dishes were incubated at 37 °C for 4–6 weeks. Based on the morphologic characteristics of *Nocardiopsis* spp. described previously^27^, colonies were picked and checked by light microscopy (BH-2; Olympus). Candidate strains were purified on inorganic salts-starch agar supplemented with 5% (w/v) NaCl^27^ and cultivated using the ISP4 medium (Difco Laboratories, Detroit, Mich) at 37 °C for four weeks^29^. Genomic DNA of the obtained strains was extracted and 16S rRNA genes were PCR amplified^19^. PCR amplification of *gyrB*, *rpoB* and *sodA* genes was performed according to the methods described previously^19^. The amplified PCR products were purified using a TaKaRa DNA fragment purification kit (Ver. 2.0) and were sequenced using an ABI 3100 automated sequencer with primers of four genes (16S rRNA, 27f and 1525r; *gyrB*, UP-1F and UP-2R; *rpoB*, MF and MR; *sodA*, Z205 and Z212)^19^ at Shanghai Sangon Biotech (Shanghai, China). The 16S rRNA gene sequences obtained from the candidate strains were compared with reference taxa via the EzTaxon-e database^52^. The sequences similarity levels were calculated between the candidate strains and their related *Nocardiopsis* taxa in the EzTaxon-e database^52^.

### Phylogenetic analysis of isolated *Nocardiopsis* strains

Multiple alignments and genetic distance calculations were carried out by using CLUSTAL_X^53^ after retrieving the reference sequences of *Nocardiopsis* type strains from the EzTaxon-e database. The pair-wise similarities between *Nocardiopsis* strains were calculated by the software package MEGA 4.0^54^. OTU classification was performed using DOTUR appliying a 98.5% 16S rRNA sequence similarity cut-off^26^. The 98.5% identity of 16S rRNA gene sequences corresponded to 70% of DNA-DNA relatedness, which was widely used as the cutoff value for species definition in prokaryotes^26^. Reference sequences were retrieved from NCBI (National Center for Biotechnology Informatics, http://www.ncbi.nlm.nih.gov) with BLAST (Basic Local Alignment Search Tool, http://blast.ncbi.nlm.nih.gov/Blast.cgi).

After designation of OTUs, phylogenies of the four investigated genes (16S rRNA, *gyrB*, *rpoB* and *sodA*) were constructed by using PhyML 3.0^55^ with maximum-likelihood^56^. Bootstrap analysis was used to evaluate the stability of tree topology by resampling 1000 times^57^. Subsequently, a cluster within an OTU of the 16S rRNA gene phylogenetic trees was defined as a phylotype. Plus, a cluster within an OTU of the *gyrB*, *rpoB* and *sodA* gene phylogenies was nominated as one genotype.

In order to differentiate between OTUs, T-test was performed to analyze pair-wise divergences of genetic distances among different strains using the Vegan package of the R software version 3.0.2. In order to study the biogeographic pattern of *Nocardiopsis*, 16S rRNA, *gyrB*, *rpoB* and *sodA* gene sequences were assigned with allele numbers and multi-locus sequence types (STs) of concatenated sequences according to the multi-locus sequence typing (MLST) web site (www.mlst.net). Phylogenies of the concatenated sequences of four investigated genes were constructed with Bayesian inference^55^ by using the PhyML 1.8.3 software with maximum-likelihood method^56^ and Mr Bayes-3.1.2^55^. Bootstrap analysis was used to evaluate the stability of tree topology by resampling 1000 times^57^.

### Biostatistic and bioinformatic analyses on the biogeographic patterns of *Nocardiopsis* strains

In order to assess the impact of spatial distances on *Nocardiopsis* strains’ dispersal, the correlations between Nei’s unbiased genetic distances of the four genes (16S rRNA, *gyrB*, *rpoB* and *sodA*) and their corresponding geographic distances were analyzed using Mantel tests implemented in the NTSYS package^58^. Additionally, the relationship between differentiation in sediments geochemistry and variations of endemic genotypes of *gyrB*, *rpoB* and *sodA* among eight sampling sites were analyzed by simple Mantel test and Canonical Correlation Analysis (CCA) with the R program^50^. The maximum-likelihood method of Yang^59^, implemented in the codeml program from the PAML package, was applied to analyze the effects of environmental forces on adaptive evolution of *Nocardiopsis* strains^59^,^60^. Six models were used to detect positive environmental selection upon evolution of the *Nocardiopsis* strains. Each model allows for various dN/dS ratios ω among sites, including the simplest model (M0 or one-ratio model), the ‘nearly neutral’ model (Mla), the positive selection model (M2a), the discrete Model M3, Model M7 (*β*), and the optimum positively selective Model M_8_. In addition, the protein prediction server (https://www.predictprotein.org)^61^ was used to map the residues under positive environmental selection to molecular function ontology of three proteins (the B subunit of DNA gyrase, the *β* subunit of RNA polymerase and A subunit of superoxide dismutase).

## Additional Information

**How to cite this article**: He, S.-T. *et al.* Biogeography of *Nocardiopsis* strains from hypersaline environments of Yunnan and Xinjiang Provinces, western China. *Sci. Rep.* **5**, 13323; doi: 10.1038/srep13323 (2015).

## Supplementary Material

Supplementary Information

## Figures and Tables

**Table 1:** Geographic information for sampling sites and numbers of sequence types specific to a sampling site, a habitat, and a region.

Sampling Sites	Latitude (N) and Longitude (E)	STs of concatenated sequences (Bootstrap values>80%)
Yunnan region		17
HJ1	25° 22.366′ N, 101° 44.568′ E	5
HJ2	25° 23.567′ N, 101° 45.105′ E	3
JC1	22° 35.069′ N, 101° 50.034′ E	4
JC2	22° 36.069′ N, 101° 52.034′ E	5
Xinjiang region		17
AK1	42° 29′ N, 89° 22′ E	4
AK2	42° 29′ N, 89° 23′ E	5
QJJ1	43° 26′ N, 91° 38′ E	6
QJJ2	43° 26′ N, 91° 38.6′ E	2

QJJ denotes Qijiaojing sampling sites, AK denotes Aydingkol sampling sites, JC denotes Jiangcheng sampling sites, and HJ denotes Heijing sampling sites.

**Table 2:** Simple Mantel test for genotypes of five *Nocardiopsis* OTUs in this study.

environmental factor	*gyrB* genotypes		*rpoB* genotypes		*sodA* genotypes	
	r	p	r	p	r	P
Cl^−^ (ppm)	0.536	0.046	0.594	0.047	0.087	0.486
Ca^2+^ (ppm)	0.636	0.026	0.694	0.047	0.587	0.046
Mg^2+^ (ppm)	0.736	0.028	0.540	0.032	0.527	0.035
Na^+^ (ppm)	0.739	0.033	0.506	0.046	0.579	0.0036
Fe^2/3+^ (ppm)	0.531	0.054	0.497	0.055	0.578	0.019
Mn^2+^ (ppm)	0.089	0.334	0.645	0.035	0.537	0.044
Cu^2+^ (ppm)	0.601	0.043	0.590	0.038	0.502	0.042
Zn^2+^ (ppm)	0.245	0.113	0.322	0.051	0.643	0.038

*r*^*2*^ is the correlation value; positive or negative values reflect the type of relationship between the two matrices, while p is the probability associated with *r*^*2*^. *P* values are significant if *P* is <0.05 (boldface).
