# Supplementary material for: Biogeography of Nocardiopsis strains from hypersaline environments of Yunnan and Xinjiang Provinces, western China
Source: Sci Rep. 2015 Aug 20;5:13323. doi: 10.1038/srep13323 (PMC4542603; doi:10.1038/srep13323)
Supplement: Supplementary Information [file srep13323-s1.doc]

**Biogeography of *Nocardiopsis* strains from hypersaline environments of Yunnan and Xinjiang Provinces, western China**

Song-Tao He^1,2†^, Xiao-Yang Zhi^2†^, Hongchen Jiang^3^*, Ling-Ling Yang^2^, Jin-Yuan Wu^2^, Yong-Guang Zhang^4^, Wael N.Hozzein^5^ & Wen-Jun Li^1,2,4*^

^1^State Key Laboratory of Biocontrol, Key Laboratory of Biodiversity Dynamics and Conservation of Guangdong Higher Education Institutes, College of Ecology and Evolution, Sun Yat-Sen University, Guangzhou, 510275, China

^2^Key Laboratory of Microbial Diversity in Southwest China, Ministry of Education, Yunnan Institute of Microbiology, Yunnan University, Kunming, 650091, China

^3^State Key Laboratory of Biogeology and Environmental Geology, China University of Geosciences, Wuhan 430074, China

^4^Key Laboratory of Biogeography and Bioresource in Arid Land, Chinese Academy of Science, Xinjiang Institute of Ecology and Geography, Chinese Academy of Sciences, Ürűmqi 830011, China

^5^Bioproducts Research Chair (BRC), College of Science, King Saud University, Riyadh 11451, Kingdom of Saudi Arabia

**Correspondence:**

**Wen-jun Li:** liwenjun3@mail.sysu.edu.cn, 86-20-84111727

**Hongchen Jiang:** [jiangh@cug.edu.cn](mailto:jiangh@cug.edu.cn), 86-27-67883452

**Table S1.** Geographic distance (kilometers) among the sampling sites in this study

| sample sites | Subtropical monsoon climate | | | | Temperate and arid climate | | | |
| --- | --- | --- | --- | --- | --- | --- | --- | --- |
|  | HJ1 | HJ2 | JC1 | JC2 | AK1 | AK2 | QJJ1 | QJJ2 |
| HJ1 | 0 | 2.4 | 565 | 561 | 4433 | 4427 | 3353 | 3264 |
| HJ2 | 2.4 | 0 | 569km | 568 | 4435 | 4429 | 3357 | 3267 |
| JC1 | 565 | 569 | 0 | 12.7 | 4461 | 4465 | 4144 | 4143 |
| JC2 | 561 | 568 | 12.7 | 0 | 4458 | 4460 | 4140 | 4138 |
| AK1 | 4433 | 4435 | 4461 | 4458 | 0 | 5 | 140 | 130 |
| AK2 | 4427 | 4429 | 4458 | 4455 | 5 | 0 | 126 | 112 |
| QJJ1 | 3353 | 3357 | 4144 | 4140 | 140 | 126 | 0 | 0.5 |
| QJJ2 | 3264 | 3267 | 4143 | 4138 | 130 | 112 | 0.5 | 0 |

**Table S2.** Major ions and trace elements of sediment samples from eight sampling sites in Yunnan and Xinjiang Provinces, western China. QJJ and AK indicate the Qijiaojing and Aydingkol sampling sites in Xinjiang Province, respectively; and JC and HJ indicate the Jiangcheng and Heijing sampling sites in Yunnan province, respectively. Abbreviation of the sample names in the following tables and figures are same as Table S1 unless specified otherwise.

| Major ions and  trace elements of  sediment samples | Sampling sites | | | | | | | |
| --- | --- | --- | --- | --- | --- | --- | --- | --- |
|  | HJ1 | HJ2 | JC1 | JC2 | AK1 | AK2 | QJJ1 | QJJ2 |
| PH | 6 | 6.5 | 6.8 | 6.4 | 8.13 | 8.06 | 8.47 | 7.8 |
| Cl^-^ (ppm) | 11.7 | 13.6 | 10.4 | 11.6 | 56.1 | 63.7 | 13.2 | 11.6 |
| Ca^2+^(ppm) | 6.4 | 8.7 | 9.8 | 10.7 | 18.8 | 27.4 | 10. 6 | 10.9 |
| Mg^2+^(ppm) | 1.3 | 1.0 | 2.4 | 3.2 | 4.0 | 4.9 | 4.4 | 3.3 |
| K^+^(ppm) | 0.5 | 0.7 | 0.1 | 0.2 | 0.2 | 0.2 | 0.1 | 0.1 |
| Na^+^(ppm) | 13.0 | 10.7 | 30.9 | 40.7 | 54.9 | 68.4 | 21.6 | 15.4 |
| Fe^2/3+^(ppm) | 10.7 | 11.5 | 16.5 | 13.5 | 8. 7 | 7.9 | 5.2 | 4.3 |
| Mn^2+^(ppm) | 4.3 | 2.1 | 4.0 | 2.8 | 10.9 | 11.0 | 6.8 | 7.9 |
| Cu^2+^(ppm) | 0.4 | 1.4 | 1.1 | 1.2 | 0.1 | 0.1 | 0.1 | 0.1 |
| Zn^2+^(ppm) | 0.9 | 1.0 | 0.7 | 0.9 | 0.1 | 0.1 | 0.1 | 0.1 |
| Salinity (%) | 3.0 | 4.0 | 0.4 | 0.6 | 11.7 | 10.0 | 7.8 | 9.4 |
| Total N | 12.6 | 10.4 | 35.9 | 20.4 | 15.6 | 16.5 | 12.0 | 12.3 |
| Total P | 1.2 | 4.2 | 2.6 | 3.2 | 0.8 | 0.6 | 0.9 | 1.0 |

**Table S3.** OTUs designation of the *Nocardiopsis* strains in this study (Each of the identified OTUs was named after the species names of their closely related *Nocardiopsis* type strains).

| Strain | Isolation site | Most closet relative *Nocardiopsis* type strain | OTU designation | 16S rRNA similarities (%) |
| --- | --- | --- | --- | --- |
| YIM 90026 | AK1 | *Nocardiopsis terrae* YIM 90022 | *Nocardiopsis terrae* | 98.5 |
| YIM 90009 | AK1 | *Nocardiopsis terrae* YIM 90022 | *Nocardiopsis terrae* | 99.5 |
| YIM 90039 | AK1 | *Nocardiopsis terrae* YIM 90022 | *Nocardiopsis terrae* | 98.5 |
| YIM 90034 | AK1 | *Nocardiopsis terrae* YIM 90022 | *Nocardiopsis terrae* | 99.5 |
| YIM 90036 | AK1 | *Nocardiopsis terrae* YIM 90022 | *Nocardiopsis terrae* | 100.0 |
| YIM 90006 | AK1 | *Nocardiopsis xinjiangensis* YIM 90004 | *Nocardiopsis xinjiangensis* | 99.0 |
| YIM 90022  (Type strain) | AK1 | *Nocardiopsis terrae* YIM 90022 | *Nocardiopsis xinjiangensis* | 100.0 |
| YIM 90004  (Type strain) | AK1 | *Nocardiopsis xinjiangensis* YIM 90004 | *Nocardiopsis terrae* | 100.0 |
| YIM 28A4  (Type strain) | AK1 | *Nocardiopsis quinghaiensis* YIM 28A4 | *Nocardiopsis quinghaiensis* | 100.0 |
| YIM 90268 | AK2 | *Nocardiopsis aegyptia* DSM 44442 | *Nocardiopsis aegyptia* | 98.6 |
| YIM 90252 | AK2 | *Nocardiopsis aegyptia* DSM 44442 | *Nocardiopsis aegyptia* | 98.7 |
| YIM 90204 | AK2 | *Nocardiopsis aegyptia* DSM 44442 | *Nocardiopsis aegyptia* | 99.6 |
| YIM 90276 | AK2 | *Nocardiopsis aegyptia* DSM 44442 | *Nocardiopsis aegyptia* | 98.6 |
| YIM 90290 | AK2 | *Nocardiopsis aegyptia* DSM 44442 | *Nocardiopsis aegyptia* | 100.0 |
| YIM 90295 | AK2 | *Nocardiopsis aegyptia* DSM 44442 | *Nocardiopsis aegyptia* | 99.0 |
| YIM 90280 | AK2 | *Nocardiopsis aegyptia* DSM 44442 | *Nocardiopsis aegyptia* | 99.6 |
| YIM 90213 | AK2 | *Nocardiopsis aegyptia* DSM 44442 | *Nocardiopsis aegyptia* | 99.0 |
| YIM 90210 | AK2 | *Nocardiopsis aegyptia* DSM 44442 | *Nocardiopsis aegyptia* | 98.6 |
| YIM 90325 | AK2 | *Nocardiopsis dassonvillei* DSM 40465 | *Nocardiopsis terrae* | 98.6 |
| YIM 90270 | AK2 | *Nocardiopsis terrae* YIM 90022 | *Nocardiopsis terrae* | 98.6 |
| YIM 90287 | AK2 | *Nocardiopsis terrae* YIM 90022 | *Nocardiopsis terrae* | 98.9 |
| YIM 90267 | AK2 | *Nocardiopsis terrae* YIM 90022 | *Nocardiopsis terrae* | 99.0 |
| YIM 90214 | AK2 | *Nocardiopsis terrae* YIM 90022 | *Nocardiopsis terrae* | 98.6 |
| YIM 90211 | AK2 | *Nocardiopsis terrae* YIM 90022 | *Nocardiopsis terrae* | 98.6 |
| YIM 90222 | AK2 | *Nocardiopsis terrae* YIM 90022 | *Nocardiopsis terrae* | 100.0 |
| YIM 90284 | AK2 | *Nocardiopsis terrae* YIM 90022 | *Nocardiopsis terrae* | 99.0 |
| YIM 90294 | AK2 | *Nocardiopsis quinghaiensis* YIM 28A4 | *Nocardiopsis quinghaiensis* | 99.6 |
| YIM 90324 | AK2 | *Nocardiopsis quinghaiensis* YIM 28A4 | *Nocardiopsis quinghaiensis* | 99.0 |
| YIM 93654 | QJJ1 | *Nocardiopsis aegyptia* DSM 44442 | *Nocardiopsis aegyptia* | 98.6 |
| YIM 93826 | QJJ1 | *Nocardiopsis aegyptia* DSM 44442 | *Nocardiopsis aegyptia* | 99.0 |
| YIM 93883 | QJJ1 | *Nocardiopsis aegyptia* DSM 44442 | *Nocardiopsis aegyptia* | 98.6 |
| YIM 93869 | QJJ1 | *Nocardiopsis dassonvillei* DSM 40465 | *Nocardiopsis dassonvillei* | 98.9 |
| YIM 93872 | QJJ1 | *Nocardiopsis dassonvillei* DSM 40465 | *Nocardiopsis dassonvillei* | 98.6 |
| YIM 93873 | QJJ1 | *Nocardiopsis dassonvillei* DSM 40465 | *Nocardiopsis dassonvillei* | 98.6 |
| YIM 93876 | QJJ1 | *Nocardiopsis dassonvillei* DSM 40465 | *Nocardiopsis dassonvillei* | 98.9 |
| YIM 93586 | QJJ1 | *Nocardiopsis terrae* YIM 90022 | *Nocardiopsis terrae* | 99.0 |
| YIM 93578 | QJJ1 | *Nocardiopsis terrae* YIM 90022 | *Nocardiopsis terrae* | 98.6 |
| YIM 93834 | QJJ1 | *Nocardiopsis terrae* YIM 90022 | *Nocardiopsis terrae* | 98.6 |
| YIM 93569 | QJJ1 | *Nocardiopsis quinghaiensis* YIM 28A4 | *Nocardiopsis quinghaiensis* | 100.0 |
| YIM 93889 | QJJ1 | *Nocardiopsis quinghaiensis* YIM 28A4 | *Nocardiopsis quinghaiensis* | 99.0 |
| YIM 93808 | QJJ1 | *Nocardiopsis quinghaiensis* YIM 28A4 | *Nocardiopsis quinghaiensis* | 98.6 |
| YIM 93811 | QJJ1 | *Nocardiopsis xinjiangensis* YIM 90004 | *Nocardiopsis xinjiangensis* | 98.7 |
| YIM 93828 | QJJ1 | *Nocardiopsis xinjiangensis* YIM 90004 | *Nocardiopsis xinjiangensis* | 98.6 |
| YIM 93914 | QJJ12 | *Nocardiopsis aegyptia* DSM 44442 | *Nocardiopsis aegyptia* | 100.0 |
| YIM 93915 | QJJ12 | *Nocardiopsis aegyptia* DSM 44442 | *Nocardiopsis aegyptia* | 99.0 |
| YIM 93923 | QJJ12 | *Nocardiopsis dassonvillei* DSM 40465 | *Nocardiopsis dassonvillei* | 98.6 |
| YIM 93926 | QJJ12 | *Nocardiopsis quinghaiensis* YIM 28A4 | *Nocardiopsis quinghaiensis* | 98.6 |
| YIM 93978 | QJJ12 | *Nocardiopsis xinjiangensis* YIM 90004 | *Nocardiopsis quinghaiensis* | 98.6 |
| YIM 93988 | QJJ12 | *Nocardiopsis xinjiangensis* YIM 90004 | *Nocardiopsis quinghaiensis* | 98.6 |
| YIM 93987 | QJJ12 | *Nocardiopsis xinjiangensis* YIM 90004 | *Nocardiopsis quinghaiensis* | 100.0 |
| YIM 93958 | QJJ12 | *Nocardiopsis xinjiangensis* YIM 90004 | *Nocardiopsis quinghaiensis* | 99.0 |
| YIM 94003 | QJJ12 | *Nocardiopsis xinjiangensis* YIM 90004 | *Nocardiopsis quinghaiensis* | 98.6 |
| YIM 92607 | HJ1 | *Nocardiopsis aegyptia* DSM 44442 | *Nocardiopsis aegyptia* | 99.2 |
| YIM 92606 | HJ1 | *Nocardiopsis aegyptia* DSM 44442 | *Nocardiopsis aegyptia* | 98.6 |
| YIM 92602 | HJ1 | *Nocardiopsis aegyptia* DSM 44442 | *Nocardiopsis aegyptia* | 99.6 |
| YIM 92608 | HJ1 | *Nocardiopsis terrae* YIM 90022 | *Nocardiopsis terrae* | 98.8 |
| YIM 92609 | HJ1 | *Nocardiopsis quinghaiensis* YIM 28A4 | *Nocardiopsis quinghaiensis* | 98.6 |
| YIM 92604 | HJ1 | *Nocardiopsis xinjiangensis* YIM 90004 | *Nocardiopsis xinjiangensis* | 100.0 |
| YIM 92630 | HJ12 | *Nocardiopsis aegyptia* DSM 44442 | *Nocardiopsis aegyptia* | 99.0 |
| YIM 92620 | HJ12 | *Nocardiopsis aegyptia* DSM 44442 | *Nocardiopsis aegyptia* | 98.6 |
| YIM 92644 | HJ12 | *Nocardiopsis dassonvillei* DSM 40465 | *Nocardiopsis dassonvillei* | 98.6 |
| YIM 92625 | HJ12 | *Nocardiopsis terrae* YIM 90022 | *Nocardiopsis terrae* | 98.6 |
| YIM 92618 | HJ12 | *Nocardiopsis terrae* YIM 90022 | *Nocardiopsis terrae* | 98.6 |
| YIM 92628 | HJ12 | *Nocardiopsis xinjiangensis* YIM 90004 | *Nocardiopsis xinjiangensis* | 98.6 |
| YIM 92626 | HJ12 | *Nocardiopsis xinjiangensis* YIM 90004 | *Nocardiopsis xinjiangensis* | 100.0 |
| YIM 94157 | JC1 | *Nocardiopsis dassonvillei* DSM 40465 | *Nocardiopsis dassonvillei* | 99.0 |
| YIM 94374 | JC1 | *Nocardiopsis terrae* YIM 90022 | *Nocardiopsis terrae* | 98.9 |
| YIM 94375 | JC1 | *Nocardiopsis terrae* YIM 90022 | *Nocardiopsis terrae* | 98.6 |
| YIM 94585 | JC1 | *Nocardiopsis terrae* YIM 90022 | *Nocardiopsis terrae* | 98.7 |
| YIM 94088 | JC1 | *Nocardiopsis quinghaiensis* YIM 28A4 | *Nocardiopsis quinghaiensis* | 98.6 |
| YIM 94370 | JC1 | *Nocardiopsis xinjiangensis* YIM 90004 | *Nocardiopsis xinjiangensis* | 100.0 |
| YIM 94366 | JC1 | *Nocardiopsis aegyptia* DSM 44442 | *Nocardiopsis aegyptia* | 99.0 |
| YIM 94779 | JC12 | *Nocardiopsis aegyptia* DSM 44442 | *Nocardiopsis aegyptia* | 98.8 |
| YIM 94819 | JC12 | *Nocardiopsis aegyptia* DSM 44442 | *Nocardiopsis aegyptia* | 98.6 |
| YIM 94863 | JC12 | *Nocardiopsis aegyptia* DSM 44442 | *Nocardiopsis aegyptia* | 98.6 |
| YIM 95110 | JC12 | *Nocardiopsis aegyptia* DSM 44442 | *Nocardiopsis aegyptia* | 98.9 |
| YIM 95046 | JC12 | *Nocardiopsis terrae* YIM 90022 | *Nocardiopsis terrae* | 100.0 |
| YIM 94866 | JC12 | *Nocardiopsis terrae* YIM 90022 | *Nocardiopsis terrae* | 99.0 |
| YIM 94870 | JC12 | *Nocardiopsis quinghaiensis* YIM 28A4 | *Nocardiopsis quinghaiensis* | 98.6 |
| YIM 94850 | JC12 | *Nocardiopsis quinghaiensis* YIM 28A4 | *Nocardiopsis quinghaiensis* | 98.7 |

**Table S4. Phylotypes (based on 16S rRNA gene), genotypes (based on *gyrB*, *rpoB* and *sodA* genes), and sequence types (STs) of the studied *Nocardiopsis* spp.**

| Strains | Phylotypes/ Genotypes of the investigated genes  (Bootstrap value >50 %) | | | | STs of concatenated sequences  (Bootstrap value > 80 %) |
| --- | --- | --- | --- | --- | --- |
|  | 16S rRNA | *gyrB* | *rpoB* | *sodA* |  |
| YIM 90026 | NTX | NTX | NTX | NTX | 1 |
| YIM 90009 | NTX | NTX | NTX | NTX | 1 |
| YIM 90039 | NTX | NTX | NTX | NTX | 1 |
| YIM 90034 | NTX | NTX | NTX | NTX | 1 |
| YIM 90036 | NTX | NTX | NTX | NTX | 1 |
| YIM 90022  (Type strain) | NTX | NTX | NTX | NTX | 2 |
| YIM 90270 | NTX | NTX | NTX | NTX | 3 |
| YIM 90287 | NTX | NTX | NTX | NTX | 3 |
| YIM 90267 | NTX | NTX | NTX | NTX | 3 |
| YIM 90214 | NTX | NTX | NTX | NTX | 4 |
| YIM 90211 | NTX | NTX | NTX | NTX | 4 |
| YIM 90222 | NTX | NTX | NTX | NTX | 4 |
| YIM 93586 | NTX | NTX | NTX | NTX | 5 |
| YIM 93578 | NTX | NTX | NTX | NTX | 5 |
| YIM 93834 | NTX | NTX | NTX | NTX | 5 |
| YIM 92608 | NTY | NTY | NTY | NTY | 6 |
| YIM 92625 | NTY | NTY | NTY | NTY | 7 |
| YIM 92618 | NTY | NTY | NTY | NTY | 7 |
| YIM 94374 | NTY | NTY | NTY | NTY | 8 |
| YIM 94375 | NTY | NTY | NTY | NTY | 8 |
| YIM 94585 | NTY | NTY | NTY | NTY | 8 |
| YIM 95046 | NTY | NTY | NTY | NTY | 9 |
| YIM 94866 | NTY | NTY | NTY | NTY | 9 |
| YIM 90004 | NXX | NXX | NXX | NXX | 10 |
| YIM 90006 | NXX | NXX | NXX | NXX | 10 |
| YIM 93811 | NXX | NXX | NXX | NXX | 11 |
| YIM 93828 | NXX | NXX | NXX | NXX | 11 |
| YIM 93978 | NXX | NXX | NXX | NXX | 12 |
| YIM 93988 | NXX | NXX | NXX | NXX | 12 |
| YIM 93987 | NXX | NXX | NXX | NXX | 12 |
| YIM 93958 | NXX | NXX | NXX | NXX | 12 |
| YIM 94003 | NXX | NXX | NXX | NXX | 12 |
| YIM 92604 | NXY | NXY | NXY | NXY | 13 |
| YIM 92628 | NXY | NXY | NXY | NXY | 14 |
| YIM 92626 | NXY | NXY | NXY | NXY | 14 |
| YIM 94370 | NXY | NXY | NXY | NXY | 15 |
| YIM 90276 | NAX | NAX | NAX | NAX | 16 |
| YIM 90290 | NAX | NAX | NAX | NAX | 16 |
| YIM 90295 | NAX | NAX | NAX | NAX | 16 |
| YIM 90280 | NAX | NAX | NAX | NAX | 16 |
| YIM 90213 | NAX | NAX | NAX | NAX | 17 |
| YIM 90210 | NAX | NAX | NAX | NAX | 17 |
| YIM 93826 | NAX | NAX | NAX | NAX | 18 |
| YIM 93654 | NAX | NAX | NAX | NAX | 18 |
| YIM 93883 | NAX | NAX | NAX | NAX | 18 |
| YIM 93914 | NAX | NAX | NAX | NAX | 19 |
| YIM 93915 | NAX | NAX | NAX | NAX | 19 |
| YIM 92607 | NAY | NAY | NAY | NAY | 20 |
| YIM 92606 | NAY | NAY | NAY | NAY | 20 |
| YIM 92602 | NAY | NAY | NAY | NAY | 20 |
| YIM 92630 | NAY | NAY | NAY | NAY | 21 |
| YIM 92620 | NAY | NAY | NAY | NAY | 21 |
| YIM 94366 | NAY | NAY | NAY | NAY | 22 |
| YIM 94779 | NAY | NAY | NAY | NAY | 23 |
| YIM 94819 | NAY | NAY | NAY | NAY | 23 |
| YIM 94863 | NAY | NAY | NAY | NAY | 23 |
| YIM 95110 | NAY | NAY | NAY | NAY | 23 |
| YIM 90325 | NDX | NDX | NDX | NDX | 24 |
| YIM 93869 | NDX | NDX | NDX | NDX | 25 |
| YIM 93872 | NDX | NDX | NDX | NDX | 25 |
| YIM 93873 | NDX | NDX | NDX | NDX | 25 |
| YIM 93876 | NDX | NDX | NDX | NDX | 25 |
| YIM 93923 | NDX | NDX | NDX | NDX | 26 |
| YIM 92644 | NDY | NDY | NDY | NDY | 27 |
| YIM 94157 | NDY | NDY | NDY | NDY | 28 |
| YIM 28A4 | NQX | NQX | NQX | NQX | 29 |
| YIM 90294 | NQX | NQX | NQX | NQX | 30 |
| YIM 90324 | NQX | NQX | NQX | NQX | 30 |
| YIM 93569 | NQX | NQX | NQX | NQX | 31 |
| YIM 93808 | NQX | NQX | NQX | NQX | 31 |
| YIM 93889 | NQX | NQX | NQX | NQX | 32 |
| YIM 93926 | NQX | NQX | NQX | NQX | 32 |
| YIM 92609 | NQY | NQY | NQY | NQY | 33 |
| YIM 94088 | NQY | NQY | NQY | NQY | 34 |
| YIM 94870 | NQY | NQY | NQY | NQY | 35 |
| YIM 94850 | NQY | NQY | NQY | NQY | 35 |

Table S5. Numbers of sequences within one phylotype/genotype/ sequence type (ST) specific to a habitat or a sampling sites ( Phylotypes/genotypes/STs specific to a sampling site are Boldface)

| Gene | OTU | phylotypes / genotypes/STs | Yunnan Province | | | | Xinjiang Province | | | |
| --- | --- | --- | --- | --- | --- | --- | --- | --- | --- | --- |
|  |  |  | HJ1 | HJ2 | JC1 | JC2 | AK1 | AK2 | QJJ1 | QJJ2 |
| 16S rRNA  (Bootstrap value >50%) | *Nocardiopsis aegyptia* | NAX1 | 0 | 0 | 0 | 0 | 0 | 6 | 2 | 1 |
|  |  | NAX2 | 0 | 0 | 0 | 0 | 0 | **2** | 0 | 0 |
|  |  | NAX3 | 0 | 0 | 0 | 0 | 0 | 1 | 1 | 1 |
|  |  | NAY1 | 1 | 2 | 2 | 2 | 0 | 0 | 0 | 0 |
|  |  | NAY2 | **1** | 0 | 0 | 0 | 0 | 0 | 0 | 0 |
|  |  | NAY3 | 1 | 0 | 0 | 1 | 0 | 0 | 0 | 0 |
|  | *Nocardiopsis dassonvillei* | NDX1 | 0 | 0 | 0 | 0 | 0 | 0 | **4** | 0 |
|  |  | NDX2 | 0 | 0 | 0 | 0 | 0 | 1 | 0 | 1 |
|  |  | NDY1 | 1 | 0 | 0 | 1 | 0 | 0 | 0 | 0 |
|  | *Nocardiopsis terrae* | NTX1 | 0 | 0 | 0 | 0 | **1** | 0 | 0 | 0 |
|  |  | NTX2 | 0 | 0 | 0 | 0 | 0 | **1** | 0 | 0 |
|  |  | NTX3 | 0 | 0 | 0 | 0 | 1 | 0 | 1 | 0 |
|  |  | NTX4 | 0 | 0 | 0 | 0 | 0 | **2** | 0 | 0 |
|  |  | NTX5 | 0 | 0 | 0 | 0 | 2 | 2 | 1 | 0 |
|  |  | NTX6 | 0 | 0 | 0 | 0 | 0 | **2** | 0 | 0 |
|  |  | NTX7 | 0 | 0 | 0 | 0 | 2 | 0 | 1 | 0 |
|  |  | NTY1 | 1 | 2 | 3 | 2 | 0 | 0 | 0 | 0 |
|  | *Nocardiopsis quinghaiensis* | NQX1 | 0 | 0 | 0 | 0 | **1** | 0 | 0 | 0 |
|  |  | NQX2 | 0 | 0 | 0 | 0 | 0 | 2 | 3 | 1 |
|  |  | NQY1 | 1 | 2 | 1 | 0 | 0 | 0 | 0 | 0 |
|  | *Nocardiopsis xinjiangensis* | NXX1 | 0 | 0 | 0 | 0 | **2** | 0 | 0 | 0 |
|  |  | NXX2 | 0 | 0 | 0 | 0 | 0 | 0 | 2 | 5 |
|  |  | NXY1 | 1 | 2 | 1 | 0 | 0 | 0 | 0 | 0 |
| *gyrB*  (Bootstrap value >50%) | *Nocardiopsis aegyptia* | NAX1 | 0 | 0 | 0 | 0 | 0 | **6** | 0 | 0 |
|  |  | NAX2 | 0 | 0 | 0 | 0 | 0 | **3** | 0 | 0 |
|  |  | NAX3 | 0 | 0 | 0 | 0 | 0 | 0 | 3 | 1 |
|  |  | NAY1 | 0 | 0 | **3** | 0 | 0 | 0 | 0 | 0 |
|  |  | NAY2 | 0 | 0 | 0 | **2** | 0 | 0 | 0 | 0 |
|  |  | NAY3 | 1 | 4 | 0 | 0 | 0 | 0 | 0 | 0 |
|  | *Nocardiopsis xinjiangensis* | NXX1 | 0 | 0 | 0 | 0 | 0 | 0 | 2 | 5 |
|  |  | NXX2 | 0 | 0 | 0 | 0 | **2** | 0 | 0 | 0 |
|  |  | NXY1 | 1 | 3 | 1 | 0 | 0 | 0 | 0 | 0 |
|  | *Nocardiopsis dassonvillei* | NDX1 | 0 | 0 | 0 | 0 | 0 | 0 | 4 | 1 |
|  |  | NDX2 | 0 | 0 | 0 | 0 | 0 | **1** | 0 | 0 |
|  |  | NDY1 | 0 | 1 | 1 | 0 | 0 | 0 | 0 | 0 |
|  | *Nocardiopsis quinghaiensis* | NQX1 | 0 | 0 | 0 | 0 | 0 | 0 | 3 | 1 |
|  |  | NQX2 | 0 | 0 | 0 | 0 | 0 | **2** | 0 | 0 |
|  |  | NQX3 | 0 | 0 | 0 | 0 | **1** | 0 | 0 | 0 |
|  |  | NQY1 | 1 | 0 | 1 | 2 | 0 | 0 | 0 | 0 |
|  | *Nocardiopsis terrae* | NTX1 | 0 | 0 | 0 | 0 | 0 | 0 | **3** | 0 |
|  |  | NTX2 | 0 | 0 | 0 | 0 | 0 | **4** | 0 | 0 |
|  |  | NTX3 | 0 | 0 | 0 | 0 | 1 | 3 | 0 | 0 |
|  |  | NTX4 | 0 | 0 | 0 | 0 | **5** | 0 | 0 | 0 |
|  |  | NTY1 | 1 | 2 | 3 | 2 | 0 | 0 | 0 | 0 |
| *rpoB*  (Bootstrap value >50%) | *Nocardiopsis aegyptia* | NAX1 | 0 | 0 | 0 | 0 | 0 | **6** | 0 | 0 |
|  |  | NAX2 | 0 | 0 | 0 | 0 | 0 | **3** | 0 | 0 |
|  |  | NAX3 | 0 | 0 | 0 | 0 | 0 | 0 | 3 | 1 |
|  |  | NAY1 | 0 | 0 | 0 | **1** | 0 | 0 | 0 | 0 |
|  |  | NAY2 | 0 | 0 | 0 | **2** | 0 | 0 | 0 | 0 |
|  |  | NAY3 | 0 | 0 | 1 | 1 | 0 | 0 | 0 | 0 |
|  |  | NAY4 | **3** | 0 | 0 | 0 | 0 | 0 | 0 | 0 |
|  | *Nocardiopsis xinjiangensis* | NXX1 | 0 | 0 | 0 | 0 | 2 | 0 | 0 | 1 |
|  |  | NXX2 | 0 | 0 | 0 | 0 | 0 | 0 | 1 | 1 |
|  |  | NXX3 | 0 | 0 | 0 | 0 | 0 | 0 | 3 | 1 |
|  |  | NXY1 | 1 | 2 | 0 | 0 | 0 | 0 | 0 | 0 |
|  |  | NXY2 | 0 | 0 | **1** | 0 | 0 | 0 | 0 | 0 |
|  | *Nocardiopsis dassonvillei* | NDX1 | 0 | 0 | 0 | 0 | 0 | **1** | 0 | 0 |
|  |  | NDX2 | 0 | 0 | 0 | 0 | 0 | 0 | 4 | 1 |
|  |  | NDY1 | 0 | 1 | 1 | 0 | 0 | 0 | 0 | 0 |
|  | *Nocardiopsis quinghaiensis* | NQX1 | 0 | 0 | 0 | 0 | 1 | 2 | 0 | 0 |
|  |  | NQX2 | 0 | 0 | 0 | 0 | 0 | 0 | 3 | 1 |
|  |  | NQX3 | 0 | 0 | 0 | 0 | **1** | 0 | 0 | 0 |
|  |  | NQY1 | 1 | 0 | 1 | 2 | 0 | 0 | 0 | 0 |
|  | *Nocardiopsis terrae* | NTX1 | 0 | 0 | 0 | 0 | **4** | 0 | 0 | 0 |
|  |  | NTX2 | 0 | 0 | 0 | 0 | 0 | 2 | 0 | 0 |
|  |  | NTX3 | 0 | 0 | 0 | 0 | 1 | 0 | 2 | 0 |
|  |  | NTX4 | 0 | 0 | 0 | 0 | 0 | 0 | **2** | 0 |
|  |  | NTX5 | 0 | 0 | 0 | 0 | 0 | 0 | **1** | 0 |
|  |  | NTX6 | 0 | 0 | 0 | 0 | 0 | **2** | 0 | 0 |
|  |  | NTX7 | 0 | 0 | 0 | 0 | 1 | 3 | 0 | 0 |
|  |  | NTY1 | 1 | 1 | 1 | 1 | 0 | 0 | 0 | 0 |
|  |  | NTY2 | 2 | 1 | 0 | 0 | 0 | 0 | 0 | 0 |
|  |  | NTY3 | 0 | 0 | 0 | **1** | 0 | 0 | 0 | 0 |
| *sodA*  (Bootst-rap value >50%) | *Nocardiopsis aegyptia* | NAX1 | 0 | 0 | 0 | 0 | 0 | 0 | **1** | 0 |
|  |  | NAX2 | 0 | 0 | 0 | 0 | 0 | **4** | 0 | 0 |
|  |  | NAX3 | 0 | 0 | 0 | 0 | 0 | 0 | 2 | 2 |
|  |  | NAX4 | 0 | 0 | 0 | 0 | 0 | **4** | 0 | 0 |
|  |  | NAY1 | 0 | 0 | 0 | **3** | 0 | 0 | 0 | 0 |
|  |  | NAY2 | 0 | 0 | 1 | 1 | 0 | 0 | 0 | 0 |
|  |  | NAY3 | 1 | 2 | 0 | 0 | 0 | 0 | 0 | 0 |
|  |  | NAY4 | **1** | 0 | 0 | 0 | 0 | 0 | 0 | 0 |
|  |  | NAY5 | 0 | **1** | 0 | 0 | 0 | 0 | 0 | 0 |
|  | *Nocardiopsis xinjiangensis* | NXX1 | 0 | 0 | 0 | 0 | 0 | 0 | 0 | **4** |
|  |  | NXX2 | 0 | 0 | 0 | 0 | 0 | 0 | **2** | 0 |
|  |  | NXX3 | 0 | 0 | 0 | 0 | 0 | 0 | 0 | **1** |
|  |  | NXX4 | 0 | 0 | 0 | 0 | **2** | 0 | 0 | 0 |
|  |  | NXY1 | 1 | 2 | 0 | 0 | 0 | 0 | 0 | 0 |
|  |  | NXY2 | 0 | 0 | **1** | 0 | 0 | 0 | 0 | 0 |
|  | *Nocardiopsis dassonvillei* | NDX1 | 0 | 0 | 0 | 0 | 0 | **1** | 0 | 0 |
|  |  | NDX2 | 0 | 0 | 0 | 0 | 0 | 0 | 4 | 1 |
|  |  | NDY1 | 0 | **1** | 0 | 0 | 0 | 0 | 0 | 0 |
|  |  | NDY2 | 0 | 0 | **1** | 0 | 0 | 0 | 0 | 0 |
|  | *Nocardiopsis quinghaiensis* | NQX1 | 0 | 0 | 0 | 0 | 1 | 2 | 0 | 0 |
|  |  | NQX2 | 0 | 0 | 0 | 0 | 0 | 0 | 3 | 1 |
|  |  | NQY1 | 0 | 0 | 1 | 2 | 0 | 0 | 0 | 0 |
|  |  | NQY2 | **1** | 0 | 0 | 0 | 0 | 0 | 0 | 0 |
|  | *Nocardiopsis terrae* | NTX1 | 0 | 0 | 0 | 0 | **4** | 0 | 0 | 0 |
|  |  | NTX2 | 0 | 0 | 0 | 0 | **2** | 0 | 0 | 0 |
|  |  | NTX3 | 0 | 0 | 0 | 0 | 0 | **3** | 0 | 0 |
|  |  | NTX4 | 0 | 0 | 0 | 0 | 0 | **2** | 0 | 0 |
|  |  | NTX5 | 0 | 0 | 0 | 0 | 0 | **2** | 0 | 0 |
|  |  | NTX6 | 0 | 0 | 0 | 0 | 0 | 0 | **3** | 0 |
|  |  | NTY1 | 0 | **1** | 0 | 0 | 0 | 0 | 0 | 0 |
|  |  | NTY2 | 0 | 0 | 0 | **2** | 0 | 0 | 0 | 0 |
|  |  | NTY3 | 0 | 0 | 1 | 2 | 0 | 0 | 0 | 0 |
|  |  | NTY4 | 0 | **1** | 0 | 0 | 0 | 0 | 0 | 0 |
|  |  | NTY5 | **1** | 0 | 0 | 0 | 0 | 0 | 0 | 0 |
| MLST  (Bootstrap value >50%) | *Nocardiopsis terrae* | 1 | 0 | 0 | 0 | 0 | **5** | 0 | 0 | 0 |
|  |  | 2 | 0 | 0 | 0 | 0 | **1** | 0 | 0 | 0 |
|  |  | 3 | 0 | 0 | 0 | 0 | 0 | **3** | 0 | 0 |
|  |  | 4 | 0 | 0 | 0 | 0 | 0 | **3** | 0 | 0 |
|  |  | 5 | 0 | 0 | 0 | 0 | 0 | 0 | **3** | 0 |
|  |  | 6 | 0 | 0 | **1** |  | 0 | 0 | 0 | 0 |
|  |  | 7 | 0 | 0 | 0 | **2** | 0 | 0 | 0 | 0 |
|  |  | 8 | **3** | 0 | 0 | 0 | 0 | 0 | 0 | 0 |
|  |  | 9 | 0 | **2** | 0 | 0 | 0 | 0 | 0 | 0 |
|  | *Nocardiopsis xinjiangensis* | 10 | 0 | 0 | 0 | 0 | **2** | 0 | 0 | 0 |
|  |  | 11 | 0 | 0 | 0 | 0 | 0 | 0 | **2** | 0 |
|  |  | 12 | 0 | 0 | 0 | 0 | 0 | 0 | **0** | **5** |
|  |  | 13 | 0 | 0 | **1** | 0 | 0 | 0 | **0** | 0 |
|  |  | 14 | 0 | 0 | 0 | **2** | 0 | 0 | **0** | 0 |
|  |  | 15 | **1** | 0 | 0 | 0 | 0 | 0 | **0** | 0 |
|  | *Nocardiopsis aegyptia* | 16 | 0 | 0 | 0 | 0 | 0 | **4** | 0 | 0 |
|  |  | 17 | 0 | 0 | 0 | 0 | 0 | **2** | 0 | 0 |
|  |  | 18 | 0 | 0 | 0 | 0 | 0 | 0 | **3** |  |
|  |  | 19 | 0 | 0 | 0 | 0 | 0 | 0 | **0** | **2** |
|  |  | 20 | 0 | 0 | 0 | 0 | 0 | 0 | 0 | 0 |
|  |  | 21 | 0 | 0 | **3** | 0 | 0 | 0 | 0 | 0 |
|  |  | 22 | 0 | 0 | 0 | **2** | 0 | 0 | 0 | 0 |
|  |  | 23 | **1** | 0 | 0 | 0 | 0 | 0 | 0 | 0 |
|  | *Nocardiopsis dassonvillei* | 24 | 0 | **4** | 0 | 0 | 0 | 0 | 0 | 0 |
|  |  | 25 | 0 | 0 | 0 | 0 | 0 | 0 | **4** | 0 |
|  |  | 26 | 0 | 0 | 0 | 0 | 0 | 0 | 0 | **1** |
|  |  | 27 | 0 | 0 | 0 | **1** | 0 | 0 | 0 | 0 |
|  |  | 28 | **1** | 0 | 0 |  | 0 | 0 | 0 | 0 |
|  | *Nocardiopsis quinghaiensis* | 29 | 0 | 0 | 0 | **1** | 0 | 0 | 0 | 0 |
|  |  | 30 | 0 | 0 | 0 |  | **2** | 0 | 0 | 0 |
|  |  | 31 | 0 | 0 | 0 | 0 | 0 | **2** | 0 | 0 |
|  |  | 32 | 0 | 0 | 0 | 0 | 0 | 0 | **2** | 0 |
|  |  | 33 | 0 | 0 | **1** | 0 | 0 | 0 | 0 | 0 |
|  |  | 34 | **1** | 0 | 0 | 0 | 0 | 0 | 0 | 0 |
|  |  | 35 | 0 | **2** | 0 | 0 | 0 | 0 | 0 | 0 |

**Table S6.** Positively selected sites log-likelihood scores, and parameter estimates for the *gyrB*, *rpoB*, *sodA* genes of the studied *Nocardiopsis* strains

| Gene | Model | Parameter estimate | Positively Selected Sites (P>90%) |
| --- | --- | --- | --- |
| *SodA* | M2a | *P2*=0.031, w2=4.323 | Asn28, **Lys30** , Lys46, **Asp51**, **Glu61**, Gly65, Pro79, Arg86, **Pro92**, Lys 98, Lys 128, Asn146, Thr169, Lys190, **Ser196** |
|  | M3 | *P3* =0.043, w2=2.323 | Asn28, **Lys30** , Lys46, **Asp51**, **Glu61**, Gly65, Pro79, Arg86, **Pro92**, Lys 98, Lys 128, Asn146, Thr169, Asn179, Asn182, Lys187, Lys190, **Ser196** |
|  | M8 | *P8*=0.041, w8*=*3.423 | Asn28, **Lys30** , Lys46, **Asp51**, **Glu61**, Gly65, **Pro92**, Lys 98  Lys 128, Asn146, Thr169, Lys190, **Ser196** |
| *gyrB* | M2a | *P2*=0.041, w2=5.433 | Met1, **Arg31**, Thr46, Asn48, **Ala55**, **Glu103**, **Lys133**, Lys160, **Lys207**, Lys210, Lys 220 |
|  | M3 | *P3*=0.045, w2=2.433 | Met1, **Arg31**, Thr46, Asn48, **Ala55**, **Glu103**, **Lys133**, Asn142, Lys149, Glu150, Pro153 , Lys160, **Lys207**, Lys210, Lys 220 |
|  | M8 | *P8*=0.032, w8=2.433 | Met1, **Arg31**, Thr46, Asn48, **Ala55**, **Glu103**, **Lys133**, Lys160, **Lys207**, Lys210 |
| *rpoB* | M2a | *P2*=0.031, w2=6.33 | **Asp8**, Asp12, Lys14, Glu23, **Arg24**, **Met25**, **Thr26**, His41, Pro50, Arg62, Gly70, **His72, Arg74**, Lys 82,  **Ser85**, Asn96, Thr97 |
|  | M3 | *P3*=0.050, w2=3.323 | **Asp8**, Asp12, Lys14, Glu23, **Arg24**, **Met25**, **Thr26**, Asp30, Asp31, Asp32, His41, Pro50, Arg62, Gly70, **His72, Arg74**, Lys 82,  **Ser85**, Asn 96, Thr97 |
|  | M8 | *P8*=0.040, w8=5.433 | **Asp8**, Lys14, Glu23, **Arg24**, **Met25**, **Thr26**, His41, Pro50, Arg62, Gly70, **His72, Arg74**, Lys 82,  **Ser85**, Asn96, Thr97 |

Three positive selection models (M2a, M3 and M8) show presence of sites under positive selection The dN/dS ratios ω for each specific model were used to detect three transcripts undergoing positive selection, ω>1 indicates residues of transcripts under positive selection. The *P* value (*P*2, *P*3, and *P*8) for each specific model was employed to estimate significance of positive selections among three transcripts．Sites inferred to be under positive selection with probabilities>0.99 are in the table, positively selected sites shared by three models are boldface.

**Table. S7.** Mapping residues under positive environmental selection onto molecular function ontology of three proteins (Subunit B of DNA gyrase, *β* subunit of bacterial RNA polymerase and subunit A of superoxide dismutase) in the molecular function ontology.

| **Metabolic networks of selected sites in the Molecular Function Ontology** | | | | |
| --- | --- | --- | --- | --- |
| **Protein** | **GO ID** | **GO Term*** | **Sites** | **Reliability (%)** |
| Subunit B of DNA gyrase (encoded by *gyrB* ) | GO:0000287 | **magnesium ion binding** | **Arg31, Ala55, Glu103, Lys133, Lys207** | **80** |
|  | GO:0008094 | DNA-dependent ATPase activities | Asn142, Lys149, GLu150, Pro153 | 85 |
|  | GO:0005515 | Protein binding | Met1, Thr46, Asn48, Lys160,Lys210 | 75 |
|  | GO:0043565 | **Sequence specific DNA binding** | **Lys207,** Lys220 | **75** |
|  | GO:0005524 | **ATP binding** | **Glu103, Lys133,** GLu150 | **65** |
| *β* subunit of bacterial RNA polymerase ( encoded by *rpoB*) | GO: 0000287 | **magnesium ion binding** | **Asp8, Arg24, Met25, Thr26, His72,Arg74, Ser85** | 80 |
|  | GO: 0005515 | protein binding | Asp12, Lys14, Glu23, Pro50, Arg62, Lys82, Asn96, Thr97 | 90 |
|  | GO: 0003729 | RNA binding | Asp12, His41, Gly70, Lys82 | 85 |
|  | GO: 0008144 | **drug binding (rifampicin resistences)** | **Asp8**, Asp30,Asp31, Asp32 | 75 |
| subunit A of superoxide dismutase (encoded by *sodA*) | GO:0000287 | **magnesium ion binding** | **Lys30, Asp51, Glu61** | 80 |
|  | GO: 0042802 | identical protein binding | Asn28, Lys46, Gly65, Pro79, Arg86, Lys128, Asn146, Thr169 | 90 |
|  | GO: 0005507 | **copper ion binding** | **Pro92**, Lys 190 | 85 |
|  | GO: 0005506 | **iron ion binding** | **Ser196** | 75 |
|  | GO: 0019825 | oxygen binding | Lys98 | 53 |
|  | GO: 0004784 | superoxide dismutase activity | Asn179, Asn182, Lys187 | 52 |

*GO: gene ontology in the metabolite; The selected sites in the both two data sets are boldface, the large data sets include 24 *Nocardiopsis* species type strains from other habitats and 78 *Nocardiopsis* strains isolated in this study, while small data sets only include 78 *Nocardiopsis* strains in this study..

**Table S8. GenBank accession numbers of the *gyrB*, *rpoB* and *sodA* gene sequences of the studied *Nocardiopsis* spp.**

| Strain No. | *gyrB* | Strain No. | *sodA* | Strain No. | *rpoB* |
| --- | --- | --- | --- | --- | --- |
| 94370 | KC479346 | 90009 | KC479424 | 90295 | KC479502 |
| 90276 | KC479347 | 90039 | KC479425 | 90252 | KC479503 |
| 93926 | KC479348 | 90022 | KC479426 | 90325 | KC479504 |
| 93808 | KC479349 | 90036 | KC479427 | 94819 | KC479505 |
| 93987 | KC479350 | 90026 | KC479428 | 90213 | KC479506 |
| 90325 | KC479351 | 92608 | KC479429 | 94374 | KC479507 |
| 90211 | KC479352 | 90222 | KC479430 | 92607 | KC479508 |
| 92604 | KC479353 | 90211 | KC479431 | 92630 | KC479509 |
| 92628 | KC479354 | 90034 | KC479432 | 92620 | KC479510 |
| 92626 | KC479355 | 93586 | KC479433 | 92606 | KC479511 |
| 93958 | KC479356 | 93834 | KC479434 | 92602 | KC479512 |
| 93978 | KC479357 | 93578 | KC479435 | 93883 | KC479513 |
| 94003 | KC479358 | 90267 | KC479436 | 90210 | KC479514 |
| 93988 | KC479359 | 90214 | KC479437 | 90280 | KC479515 |
| 93828 | KC479360 | 90270 | KC479438 | 92644 | KC479516 |
| 93811 | KC479361 | 90284 | KC479439 | 94863 | KC479517 |
| 90290 | KC479362 | 90287 | KC479440 | 94585 | KC479518 |
| 90210 | KC479363 | 94374 | KC479441 | 92608 | KC479519 |
| 90268 | KC479364 | 94375 | KC479442 | 93873 | KC479520 |
| 90252 | KC479365 | 92618 | KC479443 | 93869 | KC479521 |
| 90204 | KC479366 | 95046 | KC479444 | 93872 | KC479522 |
| 90284 | KC479367 | 94866 | KC479445 | 93578 | KC479523 |
| 90222 | KC479368 | 92625 | KC479446 | 94866 | KC479524 |
| 90026 | KC479369 | 94585 | KC479447 | 92625 | KC479525 |
| 90270 | KC479370 | 90006 | KC479448 | 94366 | KC479526 |
| 90006 | KC479371 | 93811 | KC479449 | 94779 | KC479527 |
| 90267 | KC479372 | 93828 | KC479450 | 90022 | KC479528 |
| 90214 | KC479373 | 93978 | KC479451 | 90211 | KC479529 |
| 90287 | KC479374 | 90294 | KC479452 | 90222 | KC479530 |
| 93586 | KC479375 | 90324 | KC479453 | 94157 | KC479531 |
| 93578 | KC479376 | 93988 | KC479454 | 93923 | KC479532 |
| 93834 | KC479377 | 93987 | KC479455 | 90034 | KC479533 |
| 92602 | KC479378 | 93958 | KC479456 | 90009 | KC479534 |
| 92606 | KC479379 | 94003 | KC479457 | 90039 | KC479535 |
| 92607 | KC479380 | 93808 | KC479458 | 90026 | KC479536 |
| 94863 | KC479381 | 93889 | KC479459 | 90270 | KC479537 |
| 95110 | KC479382 | 93926 | KC479460 | 90287 | KC479538 |
| 92620 | KC479383 | 93569 | KC479461 | 90284 | KC479539 |
| 92630 | KC479384 | 92604 | KC479462 | 90290 | KC479540 |
| 90009 | KC479385 | 92626 | KC479463 | 90204 | KC479541 |
| 90022 | KC479386 | 92628 | KC479464 | 94370 | KC479542 |
| 90036 | KC479387 | 94370 | KC479465 | 93988 | KC479543 |
| 90034 | KC479388 | 93873 | KC479466 | 90006 | KC479544 |
| 90039 | KC479389 | 93923 | KC479467 | 93811 | KC479545 |
| 93869 | KC479390 | 93876 | KC479468 | 93958 | KC479546 |
| 93923 | KC479391 | 93869 | KC479469 | 93828 | KC479547 |
| 94157 | KC479392 | 93872 | KC479470 | 94003 | KC479548 |
| 92644 | KC479393 | 90325 | KC479471 | 93978 | KC479549 |
| 94779 | KC479394 | 92644 | KC479472 | 93987 | KC479550 |
| 94819 | KC479395 | 94157 | KC479473 | 93834 | KC479551 |
| 94366 | KC479396 | 94088 | KC479474 | 93586 | KC479552 |
| 93872 | KC479397 | 94850 | KC479475 | 90267 | KC479553 |
| 93873 | KC479398 | 94870 | KC479476 | 90214 | KC479554 |
| 93876 | KC479399 | 92609 | KC479477 | 90036 | KC479555 |
| 93569 | KC479400 | 90204 | KC479478 | 94375 | KC479556 |
| 93889 | KC479401 | 92602 | KC479479 | 95046 | KC479557 |
| 93564 | KC479402 | 90213 | KC479480 | 92604 | KC479558 |
| 92618 | KC479403 | 90280 | KC479481 | 92626 | KC479559 |
| 92625 | KC479404 | 92620 | KC479482 | 92628 | KC479560 |
| 92608 | KC479405 | 92630 | KC479483 | 93826 | KC479561 |
| 94374 | KC479406 | 94779 | KC479484 | 93564 | KC479562 |
| 94850 | KC479407 | 94863 | KC479485 | 93915 | KC479563 |
| 93914 | KC479408 | 94819 | KC479486 | 93914 | KC479564 |
| 94866 | KC479409 | 95110 | KC479487 | 90276 | KC479565 |
| 94088 | KC479410 | 94366 | KC479488 | 90268 | KC479566 |
| 93915 | KC479411 | 92607 | KC479489 | 94850 | KC479567 |
| 94375 | KC479412 | 93915 | KC479490 | 92609 | KC479568 |
| 93883 | KC479413 | 93654 | KC479491 | 94870 | KC479569 |
| 94585 | KC479414 | 93914 | KC479492 | 94088 | KC479570 |
| 94870 | KC479415 | 93883 | KC479493 | 93876 | KC479571 |
| 92609 | KC479416 | 93826 | KC479494 | 93569 | KC479572 |
| 93826 | KC479417 | 90252 | KC479495 | 90294 | KC479573 |
| 95046 | KC479418 | 90268 | KC479496 | 90324 | KC479574 |
| 90280 | KC479419 | 90290 | KC479497 | 93926 | KC479575 |
| 90213 | KC479420 | 90210 | KC479498 | 93808 | KC479576 |
| 90295 | KC479421 | 90276 | KC479499 | 93889 | KC479577 |
| 90294 | KC479422 | 90295 | KC479500 | 92618 | KC479578 |
| 90324 | KC479423 | 92606 | KC479501 | 95110 | KC479579 |

**Table S9. GenBank accession numbers of the 16S rRNA, *gyrB*, *rpoB* and *sodA* gene sequences for the studied *Nocardiopsis* type strains**

| Type strain | 16S rRNA | *gyrB* | *sodA* | *rpoB* |
| --- | --- | --- | --- | --- |
| *N. prasina* ATCC 35940 ^T^ | X97884 | EF565425 | EF565387 | EF565435 |
| *N. listeri* DSM 40297 ^T^ | X97887 | EF565422 | EF565404 | EF565436 |
| *N. exhalans* DSM 44407 ^T^ | AY036000 | EF565417 | EF565395 | EF565440 |
| *N. xinjiangensis* YIM 90004 ^T^ | AF251709 | EF565433 | EF565393 | EF565447 |
| *N. chromatogenes* YIM 90109 ^T^ | AY619715 | EF565414 | EF565394 | EF565451 |
| *N. rhodophaeos* YIM 90096 ^T^ | AY619714 | EF565426 | EF565407 | EF565449 |
| *N. kunsanensis* DSM 44524 ^T^ | AF195412 | EF565421 | EF565390 | EF565443 |
| *N. gilva* YIM 90087 ^T^ | AY619712 | EF565418 | EF565405 | EF565448 |
| *N. halophila* DSM 44494 ^T^ | AJ421018 | EF565419 | EF565398 | EF565442 |
| *N. metallicus* DSM 44598 ^T^ | AJ420769 | EF565424 | EF565402 | EF565445 |
| *N. aegyptia* DSM 44442 ^T^ | AJ539401 | EF565411 | EF565397 | EF565441 |
| *N. alba* DSM 43377 ^T^ | X97883 | EF565412 | EF565399 | EF565437 |
| *N. lucentensis* DSM 44048 ^T^ | X97888 | EF565423 | EF565388 | EF565438 |
| *N. trehalosi* DSM 44380 ^T^ | AF105972 | EF565430 | EF565408 | EF565454 |
| *N. synnemataformans* DSM 44143 ^T^ | Y13593 | EF565429 | EF565403 | EF565452 |
| *N. tropica* DSM 44381 ^T^ | AF105971 | EF565431 | EF565396 | EF565439 |
| *N. baichengensis* YIM 90130 ^T^ | AY619716 | EF565413 | EF565392 | EF565446 |
| *N. composta* DSM 44551 ^T^ | AF360734 | EF565415 | EF565391 | EF565444 |
| *N. umidischolae* DSM 44362 ^T^ | AY036001 | EF565432 | EF565400 | EF565453 |
| *N. halotolerans* DSM 44410 ^T^ | AJ290448 | EF565420 | EF565389 | EF565455 |
| *N. rosea* YIM 90094 ^T^ | AY619713 | EF565427 | EF565406 | EF565456 |
| *N. salina* YIM 90010 ^T^ | AY373031 | EF565428 | EF565401 | EF565450 |
| *N. dassonvillei* KCTC 9190 ^T^ | X97886 | EF565416 | EF565410 | EF565457 |
| *N. alkaliphila* YIM 80379 ^T^ | AY230848 | EF565434 | EF565409 | EF565458 |

**Figure captions**

**Fig. S1.** Maximum-likelihood method-based trees of the investigated gene sequences **(**as indicated by A, B, C and D panels for 16S rRNA, *gyrB*, *rpoB* and *sodA* genes, respectively) and Bayesian phylogenetic inference of concatenated sequences of the above four genes (as indicated by the E panel). Bar, 0.02, two nucleotide substitutions per 1000nt. Solid squares (■) and upright triangles (▲) denote the studied *Nocardiopsis* strains from Qijiaojing (QJJ) and Aydingkol sampling sites (AK), respectively; Inverse triangles (▼) and diamonds (◆) denote the studied *Nocardiopsis* strains from Jiangcheng (JC) and Heijing (HJ) sampling sites in Yunnan Province, respectively.

**Fig. S1A**

**Fig. S1B**

**Fig. S1C**

**Fig. S1D**

**Fig. S1E**
